# Supplementary material for: Multiple Imputation of Missing Covariates When Using the Fine–Gray Model
Source: Stat Med. 2025 Jul 24;44(15-17):e70166. doi: 10.1002/sim.70166 (PMC12288811; doi:10.1002/sim.70166)
Supplement: Supplementary file 1 — Data S1: Supporting Inforamtion. [file SIM-44-0-s001.pdf]

Supplement to: Multiple imputation of missing covariates when using the Fine–Gray model

Edouard F. Bonneville[1], Jan Beyersmann[2], Ruth H. Keogh[3], Jonathan W. Bartlett[3], Tim P. Morris[4], Nicola Polverelli[5], Liesbeth C. de Wreede[1,6,*], and Hein Putter[1,7,*]

[1]Department of Biomedical Data Sciences, Leiden University Medical Center, The Netherlands
[2]Institute of Statistics, Ulm University, Germany
[3]Department of Medical Statistics, London School of Hygiene and Tropical Medicine, United Kingdom
[4]MRC Clinical Trials Unit at UCL, United Kingdom
[5]Unit of Bone Marrow Transplantation, Division of Hematology, Fondazione IRCCS Policlinico San Matteo di Pavia, Italy
[6]DKMS Clinical Trials Unit, Germany
[7]Mathematical Institute, Leiden University, The Netherlands
[*]Shared senior authorship

S1 Minimal code example

This is the minimal `R` code companion to section 3.4 of the main manuscript. The parameters from the simulation study scenario with $p = 0.15$, random censoring, and correctly specified Fine–Gray were used to generate the example dataset below.

```
# Load libraries
library(data.table)
library(survival)
library(kmi)
library(mice)
library(smcfcs)

# Minimal dataset
head(dat, n = 10)
```

```
   id     time D    X      Z
1   1 0.491195 0    1  0.126
2   2 0.028680 2 <NA>  1.266
3   3 0.910797 0    0 -1.571
4   4 0.217566 2    1 -0.500
5   5 0.132420 2    0  0.781
6   6 0.800913 2    0 -0.434
7   7 0.041653 2 <NA> -0.844
8   8 0.036202 1 <NA>  1.564
```

```
9    9 0.046798 0    0 -1.653
10 10 0.997413 0 <NA> -1.196
```

```r
sapply(dat, class)
```

```
       id      time         D         X         Z
"integer" "numeric"  "factor"  "factor" "numeric"
```

```r
nrow(dat)
```

```
[1] 2000
```

1. Add columns $\hat{H}_1(T)$ and $\hat{H}_2(T)$ to the original data, which are the marginal cause-specific cumulative hazards for each competing risk evaluated at an individual's event or censoring time (obtained using the Nelson–Aalen estimator).

```r
# Add cause-specific event indicators + cumulative hazards
dat$D1 <- as.numeric(dat$D == 1)
dat$D2 <- as.numeric(dat$D == 2)
dat$H1 <- nelsonaalen(data = dat, timevar = "time", statusvar = "D1")
dat$H2 <- nelsonaalen(data = dat, timevar = "time", statusvar = "D2")
```

2. Multiply impute the potential censoring time for those failing from cause 2 using {kmi}, yielding m censoring complete datasets (i.e. with "complete" V). Any completely observed covariates that are known to affect the probability of being censored should be included as predictors in the model for the censoring process. {kmi} imputes based on stratified Kaplan–Meier when Z is categorical, and based on a Cox model when Z is continuous.

```r
# 5 imputed datasets
M <- 5

# Multiply impute the censoring times
cens_imps <- kmi(
  formula = Surv(time, D != 0) ~ 1, # Additional predictors added here
  data = dat,
  etype = D,
  failcode = 1, # Specify event of interest
  nimp = M,
  #nboot = M, # Bootstrap for uncertainty in P(C > t)
  #bootstrap = TRUE
)
```

3. In each censoring complete dataset, add an additional column $\hat{\Lambda}_1(V)$. This takes the value of the marginal cumulative subdistribution hazard for cause 1 at an individual's observed or imputed subdistribution time, obtained with the Nelson–Aalen estimator based on $I(D = 1)$ and imputed V.

```r
# Preparation for covariate imputation:
# Create list of censoring complete datasets (with imputed V)
list_to_impute <- lapply(cens_imps$imputed.data, function(imp_dat) {
```

```r
  # Adjust new ordering from kmi (cause 2 individuals appended at bottom)
  dat_to_impute <- cbind(cens_imps$original.data, imp_dat)

  # Compute/add Lambda_1(V) in each imputed dataset
  dat_to_impute$Lambda1 <- nelsonaalen(
    data = dat_to_impute,
    timevar = "newtimes", # kmi naming for V
    statusvar = "D1" # I(D=1)
  )
  return(dat_to_impute)
})

# newevent is equal to I(D=1)
head(list_to_impute[[1]])
```

```
   id     time D    X      Z D1 D2         H1         H2 newtimes newevent
1   1 0.491195 0    1  0.126  0  0 0.16736459 0.55436927 0.491195        0
3   3 0.910797 0    0 -1.571  0  0 0.25761243 0.83833716 0.910797        0
8   8 0.036202 1 <NA>  1.564  1  0 0.02028935 0.09603222 0.036202        1
9   9 0.046798 0    0 -1.653  0  0 0.02606228 0.10990397 0.046798        0
10 10 0.997413 0 <NA> -1.196  0  0 0.27549886 0.87116320 0.997413        0
12 12 0.056015 0 <NA>  0.058  0  0 0.02903112 0.12350351 0.056015        0
      Lambda1
1  0.12385222
3  0.16659793
8  0.01932257
9  0.02452308
10 0.17340532
12 0.02715245
```

4. In each censoring complete dataset (each with different V and $\hat{\Lambda}_1(V)$, but same $\hat{H}_1(T)$ and $\hat{H}_2(T)$), create a single imputed dataset using the desired covariate imputation method(s).

```r
# Prepare predictor matrices for MICE using first censoring complete dataset
predmat_cs_approx <- predmat_fg_approx <- mice::make.predictorMatrix(
  data = list_to_impute[[1]]
)
predmat_cs_approx[] <- predmat_fg_approx[] <- 0

# Explicitly specify predictors to include in the imputation model
predmat_cs_approx["X", c("Z", "D1", "D2", "H1", "H2")] <- 1
predmat_fg_approx["X", c("Z", "D1", "Lambda1")] <- 1
predmat_fg_approx
```

```
         id time D X Z D1 D2 H1 H2 newtimes newevent Lambda1
id        0    0 0 0 0  0  0  0  0        0        0       0
time      0    0 0 0 0  0  0  0  0        0        0       0
D         0    0 0 0 0  0  0  0  0        0        0       0
```

```
X          0    0 0 0 1  1  0  0  0        0        0       1
Z          0    0 0 0 0  0  0  0  0        0        0       0
D1         0    0 0 0 0  0  0  0  0        0        0       0
D2         0    0 0 0 0  0  0  0  0        0        0       0
H1         0    0 0 0 0  0  0  0  0        0        0       0
H2         0    0 0 0 0  0  0  0  0        0        0       0
newtimes   0    0 0 0 0  0  0  0  0        0        0       0
newevent   0    0 0 0 0  0  0  0  0        0        0       0
Lambda1    0    0 0 0 0  0  0  0  0        0        0       0
```

```r
# Prepare the methods:
# - Approx methods: model type for X | Z, outcome
methods_approx <- mice::make.method(data = list_to_impute[[1]])

# - SMC methods: proposal model for X | Z (need to use {smcfcs} naming)
methods_smcfcs <- mice::make.method(
  data = list_to_impute[[1]],
  defaultMethod = c("norm", "logreg", "mlogit", "podds")
)
methods_smcfcs
```

```
      id     time        D        X        Z       D1       D2       H1
      ""       ""       "" "logreg"       ""       ""       ""       ""
      H2 newtimes newevent  Lambda1
      ""       ""       ""       ""
```

```r
# Impute X in each censoring complete dataset
# (parallelise this loop for speed improvements on larger data)
list_imps <- lapply(list_to_impute, function(imp_dat) {

  m <- 1
  iters <- 10 # Often upwards of 15 or 20 needed: check convergence

  imps_cs_approx <- mice(
    data = imp_dat,
    m = m,
    maxit = iters,
    method = methods_approx,
    predictorMatrix = predmat_cs_approx
  )

  imps_fg_approx <- mice(
    data = imp_dat,
    m = m,
    maxit = iters,
    method = methods_approx,
    predictorMatrix = predmat_fg_approx
  )
```

```r
  imps_cs_smc <- smcfcs(
    originaldata = imp_dat,
    smtype = "compet",
    smformula = list(
      "Surv(time, D == 1) ~ X + Z",
      "Surv(time, D == 2) ~ X + Z"
    ),
    method = methods_smcfcs,
    m = m,
    numit = iters
  )

  imps_fg_smc <- smcfcs(
    originaldata = imp_dat,
    smtype = "coxph",
    smformula = "Surv(newtimes, D1) ~ X + Z",
    method = methods_smcfcs,
    m = m,
    numit = iters
  )

  # Bring all the imputed datasets together
  imps <- rbind.data.frame(
    cbind(method = "CCA", imp_dat),
    cbind(method = "cs_smc", imps_cs_smc$impDatasets[[1]]),
    cbind(method = "cs_approx", complete(imps_cs_approx, action = 1L)),
    cbind(method = "fg_smc", imps_fg_smc$impDatasets[[1]]),
    cbind(method = "fg_approx", complete(imps_cs_approx, action = 1L))
  )
  return(imps)
})
```

5. Fit the Fine–Gray substantive model in each imputed dataset (using standard Cox software with $I(D = 1)$ and imputed V as outcome variables), and pool the estimates using Rubin's rules.

```r
# Bind everything together
dat_imps <- rbindlist(list_imps, idcol = ".imp")
dat_imps
```

```
         .imp    method    id     time      D      X      Z    D1    D2
        <int>    <char> <int>    <num> <fctr> <fctr>  <num> <num> <num>
    1:      1       CCA     1 0.491195      0      1  0.126     0     0
    2:      1       CCA     3 0.910797      0      0 -1.571     0     0
    3:      1       CCA     8 0.036202      1   <NA>  1.564     1     0
    4:      1       CCA     9 0.046798      0      0 -1.653     0     0
    5:      1       CCA    10 0.997413      0   <NA> -1.196     0     0
   ---
49996:      5 fg_approx  1992 0.319702      2      0 -2.670     0     1
```

```
49997:      5 fg_approx  1993 0.229071       2       0 -0.243      0      1
49998:      5 fg_approx  1994 1.836303       2       1 -0.366      0      1
49999:      5 fg_approx  1997 0.702380       2       0  0.283      0      1
50000:      5 fg_approx  1999 0.023554       2       1  1.377      0      1
               H1          H2 newtimes newevent     Lambda1
            <num>       <num>    <num>   <fctr>       <num>
    1: 0.16736459 0.55436927 0.491195        0 0.12385222
    2: 0.25761243 0.83833716 0.910797        0 0.16659793
    3: 0.02028935 0.09603222 0.036202        1 0.01932257
    4: 0.02606228 0.10990397 0.046798        0 0.02452308
    5: 0.27549886 0.87116320 0.997413        0 0.17340532
   ---
49996: 0.12370372 0.43826433 0.957205        0 0.17116627
49997: 0.09740419 0.35023923 0.453168        0 0.12098105
49998: 0.47538639 1.23075745 2.841599        0 0.25988878
49999: 0.21877205 0.71087168 1.170590        0 0.19454317
50000: 0.01356742 0.06584427 2.997529        0 0.26284736
```

```
# To use the usual workflow: subset one of the methods first
imps_fg_smc <- dat_imps[dat_imps$method == "fg_smc", ]

# Fit model in each imputed dataset
mods_fg_smc <- lapply(
  X = seq_len(M),
  FUN = function(m) {
    imp_m <- imps_fg_smc[imps_fg_smc$.imp == m, ]
    coxph(Surv(newtimes, D1) ~ X + Z, data = imp_m)
  }
)

# Pool results
summary(pool(mods_fg_smc))
```

```
  term  estimate  std.error statistic         df      p.value
1   X1 0.7768682 0.21722362  3.576352   9.883541 5.136286e-03
2    Z 0.4920664 0.06519244  7.547906 105.385333 1.659276e-11
```

```
# Alternative:
# Use (nested) {data.table} workflow to pool all methods simultaneously!
dat_mods <- dat_imps[, .(
  mod = list(coxph(Surv(newtimes, D1) ~ X + Z, data = .SD))
), by = c("method", ".imp")]
dat_mods
```

```
       method  .imp         mod
       <char> <int>      <list>
 1:       CCA     1 <coxph[22]>
 2:    cs_smc     1 <coxph[21]>
 3: cs_approx     1 <coxph[21]>
 4:    fg_smc     1 <coxph[21]>
```

```
 5: fg_approx     1 <coxph[21]>
 6:       CCA     2 <coxph[22]>
 7:    cs_smc     2 <coxph[21]>
 8: cs_approx     2 <coxph[21]>
 9:    fg_smc     2 <coxph[21]>
10: fg_approx     2 <coxph[21]>
11:       CCA     3 <coxph[22]>
12:    cs_smc     3 <coxph[21]>
13: cs_approx     3 <coxph[21]>
14:    fg_smc     3 <coxph[21]>
15: fg_approx     3 <coxph[21]>
16:       CCA     4 <coxph[22]>
17:    cs_smc     4 <coxph[21]>
18: cs_approx     4 <coxph[21]>
19:    fg_smc     4 <coxph[21]>
20: fg_approx     4 <coxph[21]>
21:       CCA     5 <coxph[22]>
22:    cs_smc     5 <coxph[21]>
23: cs_approx     5 <coxph[21]>
24:    fg_smc     5 <coxph[21]>
25: fg_approx     5 <coxph[21]>
       method  .imp         mod
```

```
dat_mods[, summary(pool(as.list(mod))), by = "method"]
```

```
       method   term  estimate  std.error statistic         df      p.value
       <char> <fctr>     <num>      <num>     <num>      <num>        <num>
 1:       CCA     X1 0.7781281 0.17916465  4.343089 152.067624 2.554742e-05
 2:       CCA      Z 0.4003856 0.10186017  3.930737 145.744472 1.304356e-04
 3:    cs_smc     X1 0.6980657 0.18538543  3.765483  14.973349 1.875994e-03
 4:    cs_smc      Z 0.5079436 0.06538007  7.769090  93.531830 9.965454e-12
 5: cs_approx     X1 0.6092265 0.19461615  3.130400  12.205414 8.525728e-03
 6: cs_approx      Z 0.5225790 0.06779656  7.708046  58.618467 1.775328e-10
 7:    fg_smc     X1 0.7768682 0.21722362  3.576352   9.883541 5.136286e-03
 8:    fg_smc      Z 0.4920664 0.06519244  7.547906 105.385333 1.659276e-11
 9: fg_approx     X1 0.6092265 0.19461615  3.130400  12.205414 8.525728e-03
10: fg_approx      Z 0.5225790 0.06779656  7.708046  58.618467 1.775328e-10
```

For method FG-SMC, making use of the wrapper function `smcfcs::smcfcs.finegray()` will make it easier to check convergence issues:

```
# Define methods
# .. and make sure competing event indicator is numeric
methods_smcfcs <- mice::make.method(
  data = dat,
  defaultMethod = c("norm", "logreg", "mlogit", "podds")
)
dat$D <- as.numeric(as.character(dat$D))

# Use larger number of iterations to check convergence
```

```r
imps <- smcfcs::smcfcs.finegray(
  originaldata = dat,
  smformula = "Surv(time, D) ~ X + Z",
  method = methods_smcfcs,
  cause = 1,
  m = 5,
  numit = 50,
  kmi_args = list(formula = ~ 1) # Add censoring predictors here
)
```

Check for convergence:

```r
plot(imps) +
  scale_y_continuous(
    limits = c(0, 1.25),
    breaks = seq(0, 1.25, by = 0.25)
  )
```

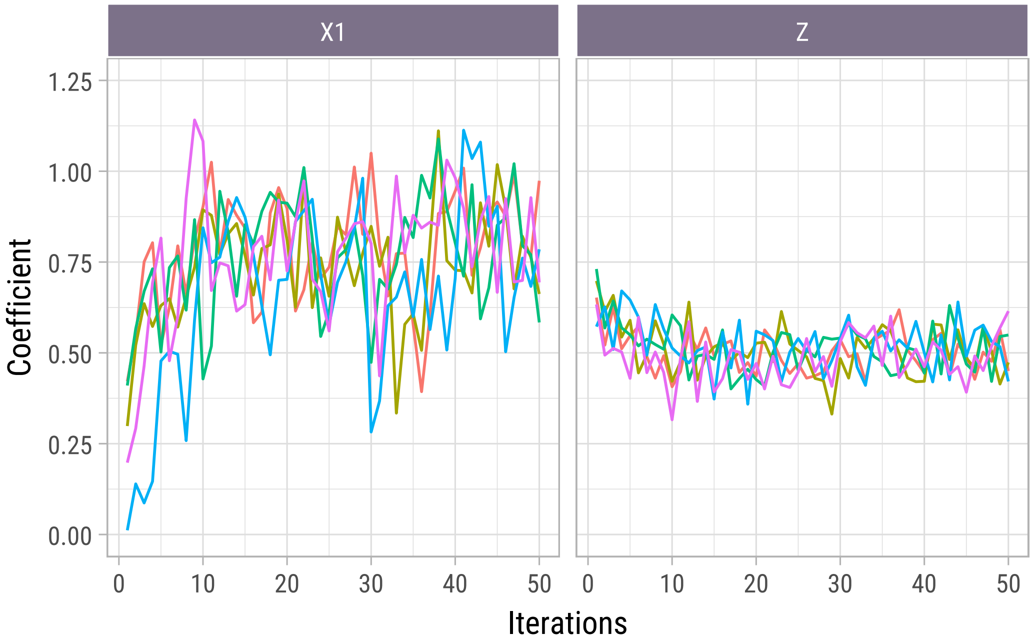

S2 Additional simulation study results

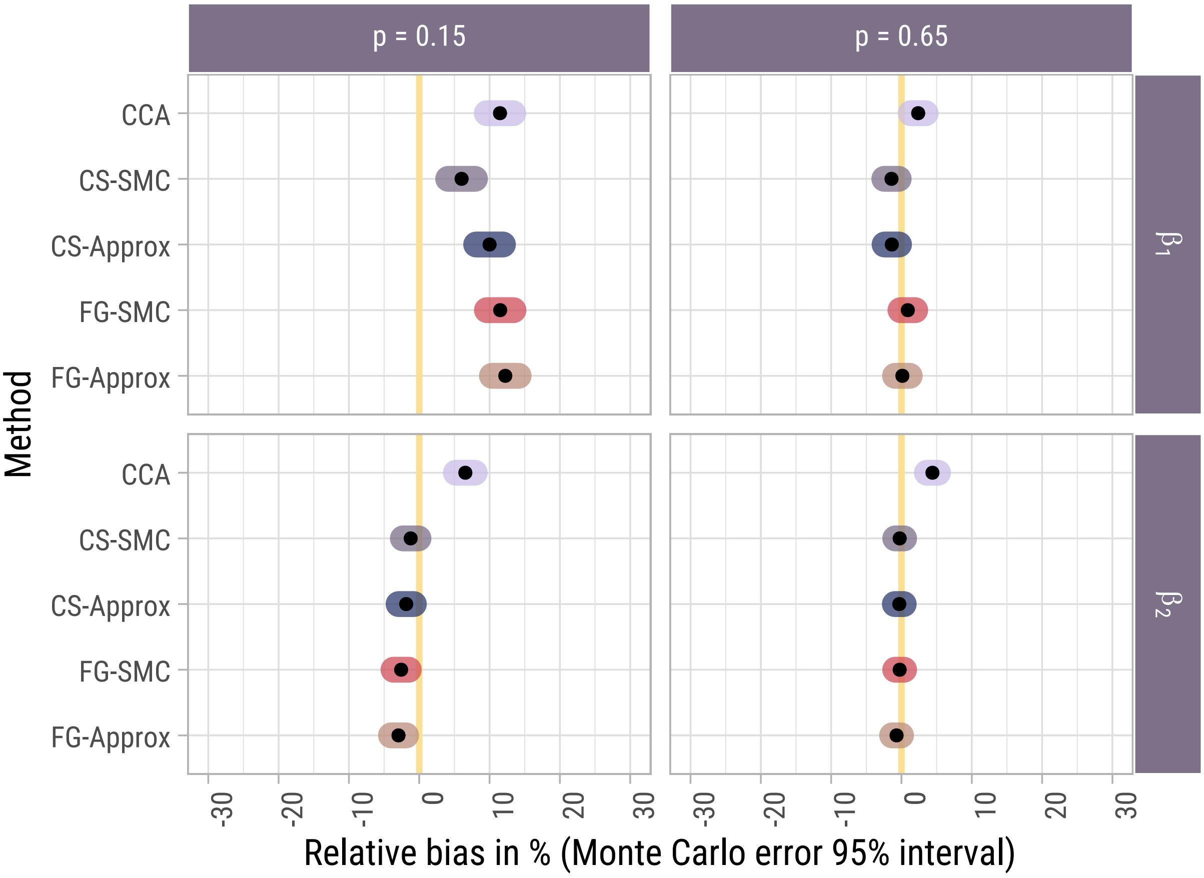

Figure 1: Relative bias (%) in estimating $\{\beta_1, \beta_2\} = \{0.75, 0.5\}$, with corresponding 95% Monte Carlo confidence interval (constructed using the standard normal approximation). These are additional simulations under the correctly specified Fine–Gray data-generating mechanism with random censoring, with both $p = 0.15$ and $p = 0.65$. The missingness in X was made to depend on the observed event time T as $\text{logit}\, P(R_X = 0 \,|\, T) = \eta_0 + \eta_1 \log(T + 1)$, with $\eta_1 = -1.5$ and η_0 chosen such that 40% of observations in X are missing.

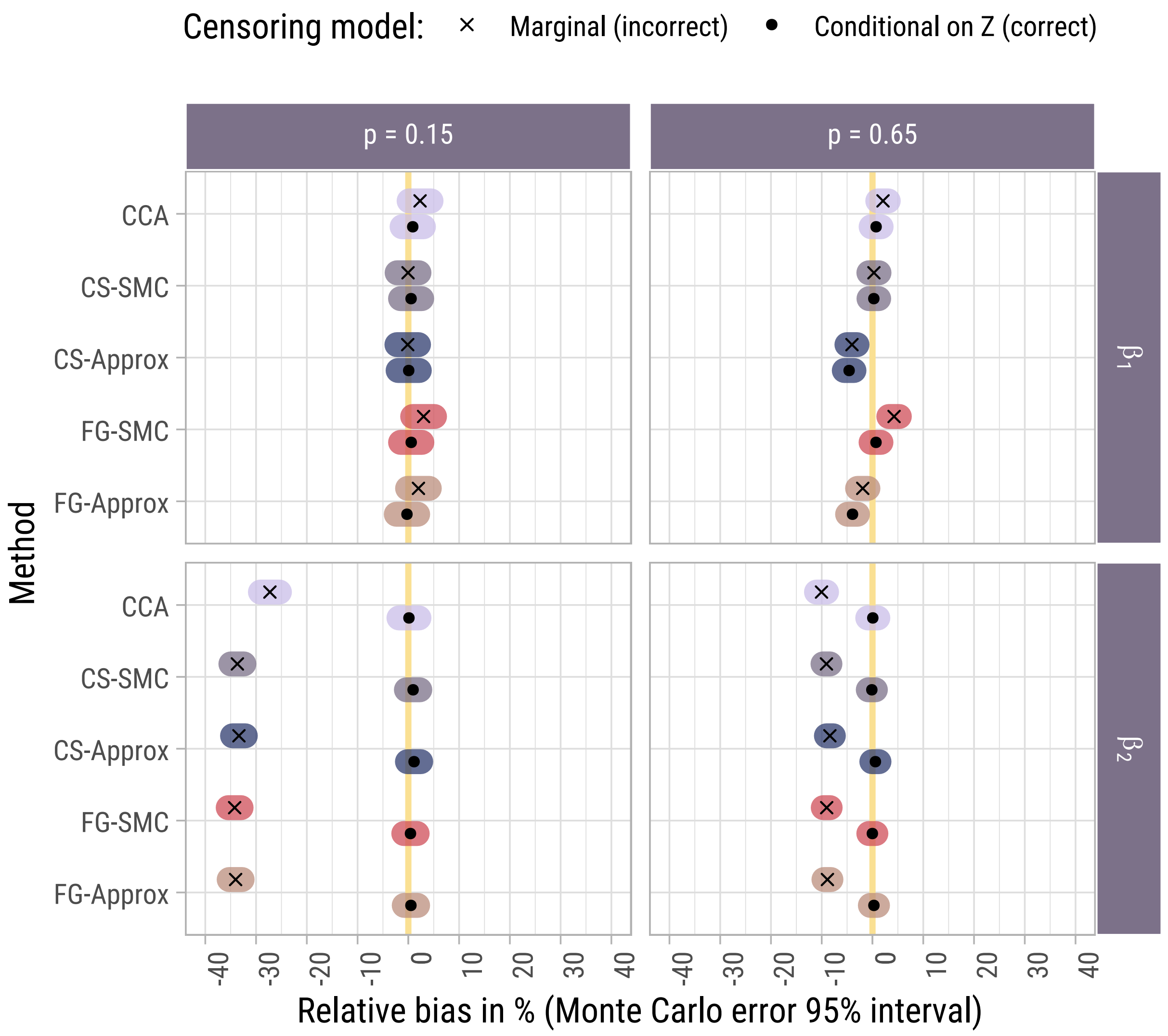

Figure 2: Relative bias (%) in estimating $\{\beta_1, \beta_2\} = \{0.75, 0.5\}$, with corresponding 95% Monte Carlo confidence interval (constructed using the standard normal approximation). These are additional simulations under the correctly specified Fine–Gray data-generating mechanism with random censoring, with both $p = 0.15$ and $p = 0.65$. The censoring was made covariate-dependent with rate $\lambda_C = 0.49e^Z$, and all covariate imputation approaches were applied after multiply imputing the potential censoring times using either a) a marginal (incorrect) Kaplan–Meier estimate of the censoring distribution; b) a Cox model for the censoring distribution, conditional on Z (correct). The missingness in X here also depended on Z.

S3 Applied data example

S3.1 Data dictionary

Table 1: Data dictionary. CMV: cytomegalovirus; HLA: human leukocyte antigen; HCT-CI: Hematopoietic stem cell transplantation-comorbidity index; MF: myelofibrosis.

Characteristic	N = 3,982
Patient age (years)	58 (52, 64)
Patient/donor CMV match	
Patient negative/Donor negative	1,142 (30%)
Other	2,715 (70%)
(Missing)	125
Donor type	
HLA identical sibling	1,183 (30%)
Other	2,795 (70%)
(Missing)	4
Hemoglobin (g/dL)	9.10 (8.10, 10.40)
(Missing)	1,873
HCT-CI risk category	
Low risk (0)	1,674 (54%)
Intermediate risk $(1-2)$	743 (24%)
High risk (≥ 3)	674 (22%)
(Missing)	891
Interval diagnosis-transplantation (years)	3 (1, 9)
Karnosfky performance score	
≥ 90	2,475 (66%)
80	986 (26%)
≤ 70	267 (7.2%)
(Missing)	254
Patient sex	
Female	1,484 (37%)
Male	2,498 (63%)
Peripheral blood (PB) blasts (%)	1.0 (0.0, 3.0)
(Missing)	2,323
Conditioning	
Standard	1,373 (35%)
Reduced	2,553 (65%)
(Missing)	56
Ruxolitinib given	
No	1,832 (66%)
Yes	931 (34%)
(Missing)	1,219
Disease subclassification	
Primary MF	2,912 (73%)
Secondary MF	1,070 (27%)
Night sweats	

No	1,256 (70%)
Yes	529 (30%)
(Missing)	2,197
T-cell depletion (in- or ev-vivo)	
No	1,012 (26%)
Yes	2,905 (74%)
(Missing)	65
Cytogenetics	
Normal	1,318 (59%)
Abnormal	910 (41%)
(Missing)	1,754
White blood cell count (WBC, x10^9/L)	7 (4, 14)
(Missing)	1,884
>10% Weight loss prior to transplantation	
No	1,329 (73%)
Yes	492 (27%)
(Missing)	2,161
Year of transplantation	2,015.0 (2,012.0, 2,018.0)

[1] Median (IQR); n (%)

S3.2 Non-parametric cumulative incidence curves

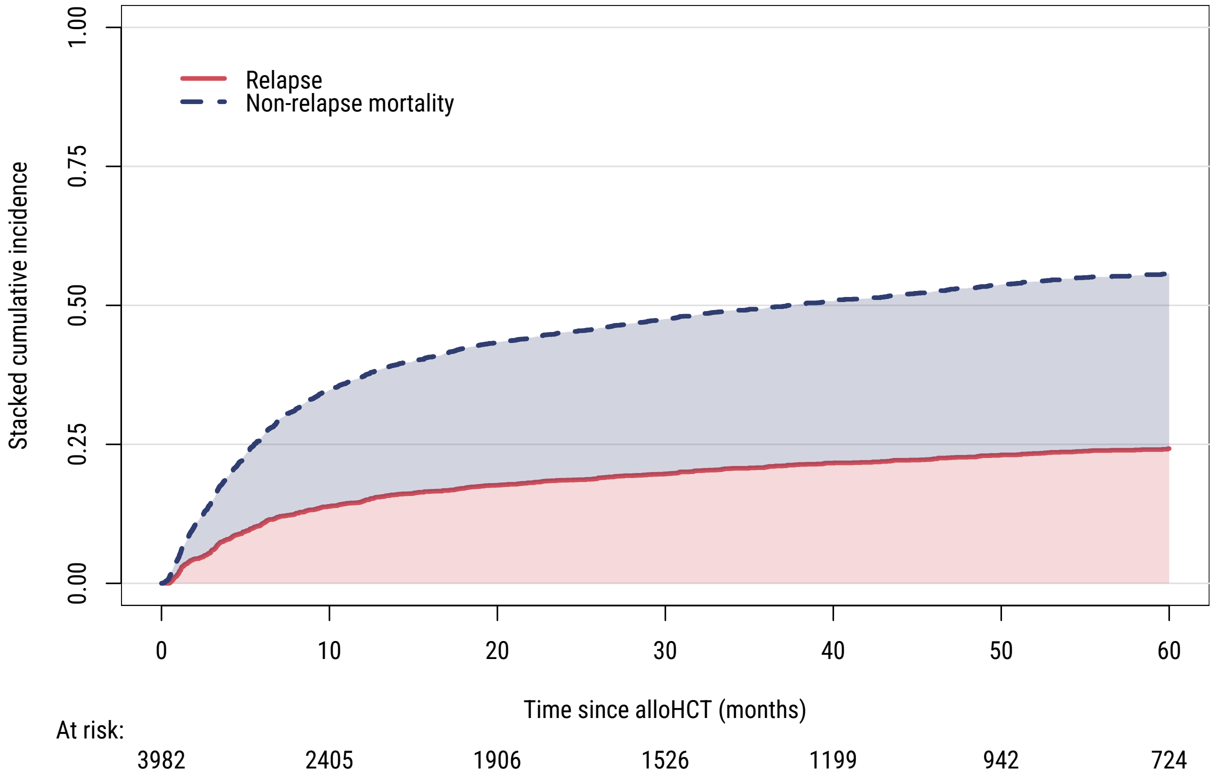

Figure 3: Stacked non-parametric cumulative incidence curves for competing relapse and non-relapse mortality, in dataset of 3982 primary and secondary myelofibrosis patients.

S3.3 Pooled regression coefficients

Table 2: Pooled log hazard ratios [log HR, 95% confidence interval] for Fine–Gray model for relapse, cause-specific Cox model for relapse, and cause-specific Cox model for non-relapse mortality (NRM).

Term + method	Relapse subdist. log HR	Relapse cause-spec. log HR	NRM cause-spec. log HR
**Conditioning: reduced**			
CCA	0.02 [-0.33, 0.36]	0.01 [-0.33, 0.35]	0 [-0.29, 0.28]
CS-SMC	0.13 [-0.02, 0.28]	0.1 [-0.05, 0.25]	-0.05 [-0.18, 0.07]
CS-Approx	0.13 [-0.02, 0.28]	0.1 [-0.05, 0.25]	-0.05 [-0.18, 0.07]
FG-SMC	0.13 [-0.02, 0.28]	0.1 [-0.05, 0.25]	-0.06 [-0.18, 0.07]
FG-Approx	0.13 [-0.03, 0.28]	0.1 [-0.06, 0.25]	-0.05 [-0.18, 0.07]
**CMV match: other**			
CCA	0.04 [-0.31, 0.4]	0.05 [-0.3, 0.41]	0.09 [-0.19, 0.37]
CS-SMC	-0.1 [-0.26, 0.05]	-0.05 [-0.2, 0.11]	0.22 [0.08, 0.36]
CS-Approx	-0.1 [-0.26, 0.05]	-0.05 [-0.2, 0.11]	0.22 [0.08, 0.36]
FG-SMC	-0.1 [-0.26, 0.05]	-0.04 [-0.2, 0.11]	0.22 [0.08, 0.36]
FG-Approx	-0.11 [-0.26, 0.05]	-0.05 [-0.2, 0.11]	0.22 [0.08, 0.35]
**Cytogenetics: abnormal**			
CCA	0.36 [0.04, 0.68]	0.37 [0.05, 0.68]	-0.08 [-0.35, 0.19]
CS-SMC	0.35 [0.15, 0.54]	0.35 [0.16, 0.54]	-0.07 [-0.23, 0.1]
CS-Approx	0.36 [0.17, 0.55]	0.35 [0.16, 0.54]	-0.08 [-0.25, 0.08]
FG-SMC	0.36 [0.17, 0.55]	0.36 [0.17, 0.54]	-0.06 [-0.21, 0.08]
FG-Approx	0.34 [0.17, 0.52]	0.34 [0.17, 0.51]	-0.07 [-0.22, 0.08]
**Donor relation: other**			
CCA	0.12 [-0.28, 0.52]	0.2 [-0.2, 0.6]	0.53 [0.18, 0.88]
CS-SMC	-0.26 [-0.41, -0.1]	-0.19 [-0.34, -0.03]	0.35 [0.21, 0.5]
CS-Approx	-0.25 [-0.41, -0.1]	-0.18 [-0.34, -0.02]	0.36 [0.21, 0.5]
FG-SMC	-0.26 [-0.41, -0.1]	-0.19 [-0.34, -0.03]	0.35 [0.2, 0.49]
FG-Approx	-0.26 [-0.41, -0.1]	-0.19 [-0.34, -0.03]	0.35 [0.2, 0.49]
**Hemoglobin (per 5 g/dL)**			
CCA	-0.38 [-0.85, 0.09]	-0.39 [-0.85, 0.08]	-0.12 [-0.49, 0.25]
CS-SMC	-0.24 [-0.51, 0.03]	-0.3 [-0.58, -0.03]	-0.19 [-0.42, 0.04]
CS-Approx	-0.25 [-0.53, 0.02]	-0.32 [-0.59, -0.06]	-0.19 [-0.41, 0.02]
FG-SMC	-0.25 [-0.51, 0.02]	-0.29 [-0.56, -0.02]	-0.08 [-0.28, 0.11]
FG-Approx	-0.23 [-0.5, 0.04]	-0.27 [-0.54, 0]	-0.09 [-0.29, 0.11]
**HCT-CI** $(1-2)$			
CCA	-0.15 [-0.53, 0.22]	-0.04 [-0.42, 0.33]	0.38 [0.08, 0.69]
CS-SMC	-0.22 [-0.42, -0.01]	-0.17 [-0.37, 0.03]	0.15 [-0.02, 0.31]
CS-Approx	-0.19 [-0.38, 0.01]	-0.14 [-0.34, 0.06]	0.15 [-0.01, 0.31]
FG-SMC	-0.22 [-0.42, -0.01]	-0.18 [-0.38, 0.02]	0.12 [-0.04, 0.28]
FG-Approx	-0.19 [-0.38, 0.01]	-0.15 [-0.35, 0.04]	0.11 [-0.05, 0.27]
**HCT-CI** (≥ 3)			
CCA	-0.27 [-0.7, 0.16]	-0.19 [-0.62, 0.23]	0.4 [0.07, 0.73]
CS-SMC	-0.07 [-0.28, 0.14]	-0.01 [-0.21, 0.2]	0.27 [0.1, 0.44]
CS-Approx	-0.08 [-0.28, 0.13]	-0.02 [-0.22, 0.18]	0.26 [0.1, 0.43]
FG-SMC	-0.06 [-0.27, 0.14]	-0.02 [-0.22, 0.19]	0.21 [0.05, 0.37]
FG-Approx	-0.08 [-0.28, 0.11]	-0.04 [-0.23, 0.16]	0.21 [0.05, 0.38]
**Interval diagnosis to alloHCT (decades)**			
CCA	0.01 [-0.24, 0.26]	0 [-0.25, 0.26]	-0.03 [-0.25, 0.19]
CS-SMC	-0.02 [-0.14, 0.09]	-0.02 [-0.14, 0.1]	0.05 [-0.05, 0.15]
CS-Approx	-0.03 [-0.14, 0.09]	-0.02 [-0.14, 0.1]	0.05 [-0.05, 0.15]
FG-SMC	-0.02 [-0.14, 0.09]	-0.02 [-0.13, 0.1]	0.05 [-0.05, 0.15]
FG-Approx	-0.02 [-0.14, 0.09]	-0.02 [-0.14, 0.1]	0.05 [-0.05, 0.15]
**Karnofsky** (80)			
CCA	-0.09 [-0.48, 0.31]	-0.08 [-0.48, 0.31]	0.04 [-0.27, 0.34]
CS-SMC	0.07 [-0.1, 0.24]	0.12 [-0.05, 0.28]	0.17 [0.03, 0.31]
CS-Approx	0.06 [-0.1, 0.23]	0.1 [-0.06, 0.27]	0.15 [0.01, 0.29]

(continued ...)

Table 2: *(continued)*

Term + method	Relapse subdist. log HR	Relapse cause-spec. log HR	NRM cause-spec. log HR
FG-SMC	0.07 [-0.09, 0.24]	0.12 [-0.05, 0.29]	0.17 [0.03, 0.31]
FG-Approx	0.07 [-0.1, 0.24]	0.12 [-0.06, 0.29]	0.17 [0.03, 0.31]
**Karnofsky** ($\leq$ 70)			
CCA	0.63 [0.15, 1.11]	0.79 [0.3, 1.28]	0.33 [-0.13, 0.79]
CS-SMC	0.44 [0.19, 0.69]	0.55 [0.3, 0.81]	0.31 [0.08, 0.53]
CS-Approx	0.42 [0.17, 0.67]	0.51 [0.26, 0.76]	0.26 [0.04, 0.49]
FG-SMC	0.44 [0.19, 0.7]	0.55 [0.29, 0.81]	0.32 [0.09, 0.54]
FG-Approx	0.43 [0.17, 0.68]	0.53 [0.28, 0.78]	0.31 [0.08, 0.53]
**Disease subclassification: secondary MF**			
CCA	-0.05 [-0.45, 0.35]	-0.02 [-0.42, 0.38]	0.07 [-0.27, 0.41]
CS-SMC	0.01 [-0.17, 0.19]	0.01 [-0.17, 0.19]	0 [-0.16, 0.15]
CS-Approx	0 [-0.18, 0.18]	0 [-0.18, 0.19]	0 [-0.16, 0.15]
FG-SMC	0 [-0.18, 0.18]	0 [-0.18, 0.18]	-0.01 [-0.16, 0.15]
FG-Approx	0 [-0.18, 0.18]	0 [-0.18, 0.18]	-0.01 [-0.16, 0.15]
**Night sweats: yes**			
CCA	-0.33 [-0.7, 0.04]	-0.4 [-0.77, -0.02]	-0.02 [-0.32, 0.27]
CS-SMC	-0.18 [-0.41, 0.05]	-0.2 [-0.44, 0.03]	-0.02 [-0.23, 0.19]
CS-Approx	-0.12 [-0.36, 0.13]	-0.14 [-0.38, 0.1]	0.03 [-0.19, 0.24]
FG-SMC	-0.17 [-0.4, 0.07]	-0.18 [-0.41, 0.05]	0.01 [-0.16, 0.19]
FG-Approx	-0.16 [-0.4, 0.07]	-0.18 [-0.42, 0.05]	0 [-0.17, 0.18]
**Patient age (decades)**			
CCA	0.1 [-0.09, 0.28]	0.13 [-0.06, 0.32]	0.13 [-0.02, 0.28]
CS-SMC	-0.03 [-0.12, 0.05]	0.01 [-0.08, 0.09]	0.21 [0.14, 0.29]
CS-Approx	-0.03 [-0.12, 0.05]	0.01 [-0.08, 0.09]	0.21 [0.14, 0.29]
FG-SMC	-0.04 [-0.12, 0.05]	0.01 [-0.08, 0.09]	0.22 [0.15, 0.3]
FG-Approx	-0.03 [-0.12, 0.05]	0.01 [-0.08, 0.09]	0.22 [0.15, 0.3]
**Patient sex: male**			
CCA	-0.24 [-0.56, 0.09]	-0.18 [-0.51, 0.15]	0.39 [0.11, 0.68]
CS-SMC	-0.1 [-0.24, 0.05]	-0.06 [-0.21, 0.09]	0.18 [0.05, 0.31]
CS-Approx	-0.1 [-0.24, 0.05]	-0.06 [-0.21, 0.09]	0.18 [0.05, 0.31]
FG-SMC	-0.09 [-0.24, 0.05]	-0.06 [-0.2, 0.09]	0.18 [0.05, 0.31]
FG-Approx	-0.1 [-0.24, 0.05]	-0.06 [-0.21, 0.08]	0.18 [0.05, 0.31]
**PB Blasts (per 5%)**			
CCA	0.16 [-0.04, 0.36]	0.17 [-0.02, 0.37]	0 [-0.18, 0.18]
CS-SMC	0.18 [0.05, 0.31]	0.18 [0.05, 0.31]	0.01 [-0.12, 0.13]
CS-Approx	0.19 [0.07, 0.31]	0.19 [0.07, 0.32]	0.01 [-0.12, 0.13]
FG-SMC	0.17 [0.04, 0.3]	0.17 [0.05, 0.3]	-0.01 [-0.12, 0.1]
FG-Approx	0.18 [0.05, 0.32]	0.18 [0.05, 0.31]	-0.02 [-0.12, 0.09]
**Ruxolitinib given: yes**			
CCA	0.08 [-0.26, 0.43]	0.08 [-0.26, 0.43]	-0.05 [-0.33, 0.23]
CS-SMC	-0.02 [-0.2, 0.17]	-0.03 [-0.22, 0.16]	-0.06 [-0.21, 0.1]
CS-Approx	0.01 [-0.19, 0.2]	-0.01 [-0.2, 0.18]	-0.05 [-0.21, 0.11]
FG-SMC	-0.02 [-0.21, 0.17]	-0.03 [-0.22, 0.16]	-0.04 [-0.19, 0.11]
FG-Approx	0 [-0.19, 0.18]	-0.01 [-0.2, 0.17]	-0.04 [-0.19, 0.11]
**T-cell depletion: yes**			
CCA	0.2 [-0.21, 0.62]	0.16 [-0.25, 0.58]	-0.23 [-0.54, 0.08]
CS-SMC	0.3 [0.13, 0.48]	0.26 [0.09, 0.44]	-0.18 [-0.32, -0.04]
CS-Approx	0.3 [0.12, 0.48]	0.26 [0.08, 0.43]	-0.19 [-0.33, -0.05]
FG-SMC	0.31 [0.13, 0.48]	0.26 [0.09, 0.44]	-0.18 [-0.31, -0.04]
FG-Approx	0.31 [0.13, 0.48]	0.26 [0.09, 0.44]	-0.18 [-0.32, -0.04]
**WBC count (log)**			
CCA	0.17 [0.02, 0.33]	0.17 [0.01, 0.33]	0.02 [-0.12, 0.15]
CS-SMC	0.17 [0.09, 0.26]	0.18 [0.09, 0.27]	0 [-0.07, 0.07]
CS-Approx	0.17 [0.08, 0.26]	0.17 [0.09, 0.26]	0 [-0.08, 0.07]
FG-SMC	0.17 [0.09, 0.26]	0.18 [0.09, 0.26]	-0.01 [-0.07, 0.05]
FG-Approx	0.17 [0.1, 0.25]	0.18 [0.1, 0.26]	-0.01 [-0.08, 0.05]

(continued ...)

Table 2: *(continued)*

Term + method	Relapse subdist. log HR	Relapse cause-spec. log HR	NRM cause-spec. log HR
**Weight loss: yes**			
CCA	0 [-0.37, 0.38]	0.05 [-0.33, 0.43]	0.17 [-0.13, 0.48]
CS-SMC	0.23 [-0.03, 0.49]	0.27 [0.01, 0.53]	0.16 [-0.05, 0.36]
CS-Approx	0.24 [0, 0.47]	0.28 [0.04, 0.51]	0.16 [-0.05, 0.36]
FG-SMC	0.23 [-0.01, 0.47]	0.24 [0.01, 0.48]	0.06 [-0.12, 0.24]
FG-Approx	0.24 [0, 0.48]	0.26 [0.02, 0.49]	0.06 [-0.14, 0.26]
**Year of alloHCT (decades)**			
CCA	-0.36 [-0.99, 0.26]	-0.41 [-1.04, 0.23]	-0.15 [-0.67, 0.37]
CS-SMC	-0.08 [-0.34, 0.18]	-0.11 [-0.37, 0.15]	-0.24 [-0.46, -0.02]
CS-Approx	-0.09 [-0.35, 0.17]	-0.12 [-0.38, 0.14]	-0.24 [-0.46, -0.02]
FG-SMC	-0.08 [-0.34, 0.17]	-0.12 [-0.37, 0.14]	-0.24 [-0.46, -0.03]
FG-Approx	-0.08 [-0.34, 0.17]	-0.11 [-0.37, 0.14]	-0.24 [-0.46, -0.03]
